# Supplementary material for: Causal Relationship between Aging and Anorexia Nervosa: A White-Matter-Microstructure-Mediated Mendelian Randomization Analysis
Source: Biomedicines. 2024 Aug 16;12(8):1874. doi: 10.3390/biomedicines12081874 (PMC11351342; doi:10.3390/biomedicines12081874)
Supplement: Supplementary file 1 [file biomedicines-12-01874-s001.zip › Supplementary Figure/Figure S4/Singe SNP plot of MR analysis for TL to ubm-b-1631.pdf]

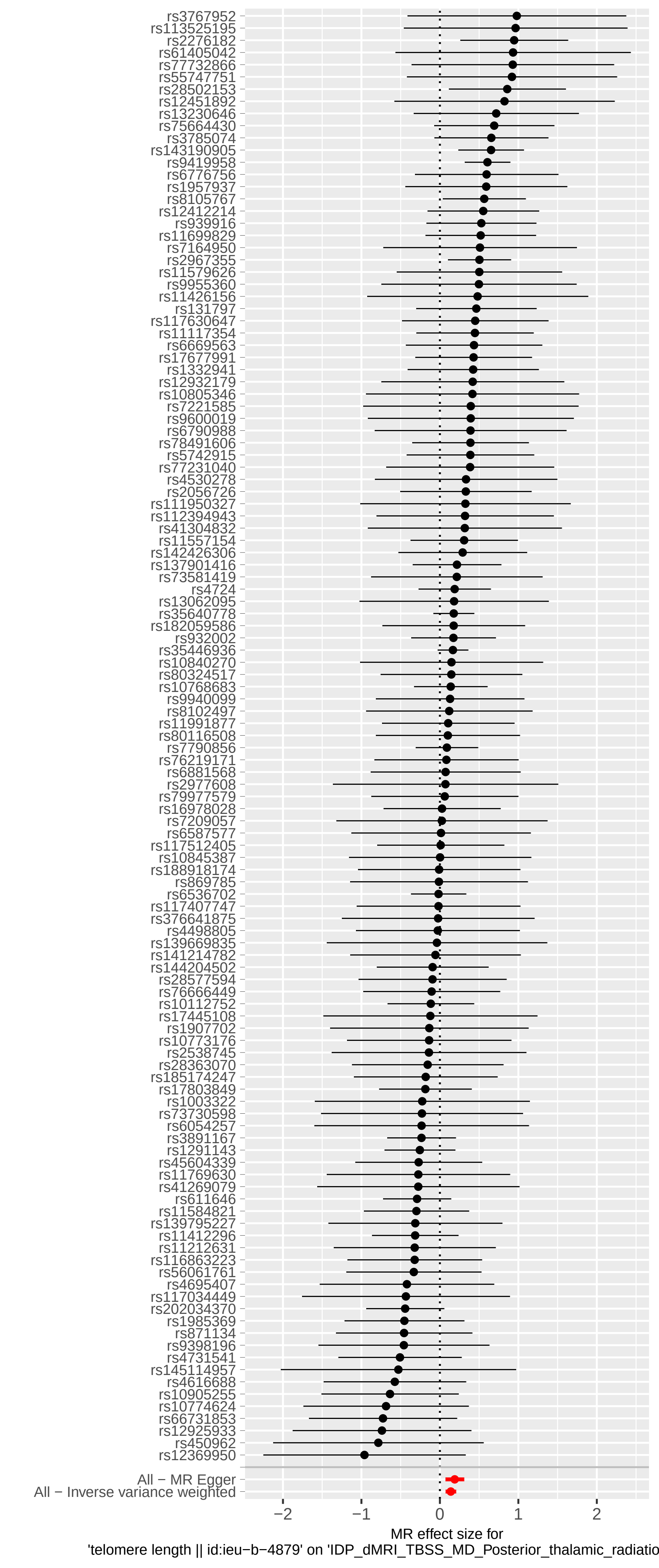
rs3767952
rs113525195
rs2276182
rs61405042
rs77732866
rs55747751
rs28502153
rs12451892
rs13230646
rs75664430
rs3785074
rs143190905
rs9419958
rs6776756
rs1957937
rs8105767
rs12412214
rs939916
rs11699829
rs7164950
rs2967355
rs11579626
rs9955360
rs11426156
rs131797
rs117630647
rs11117354
rs6669563
rs17677991
rs1332941
rs12932179
rs10805346
rs7221585
rs9600019
rs6790988
rs78491606
rs5742915
rs77231040
rs4530278
rs2056726
rs111950327
rs112394943
rs41304832
rs11557154
rs142426306
rs137901416
rs73581419
rs4724
rs13062095
rs35640778
rs182059586
rs932002
rs35446936
rs10840270
rs80324517
rs10768683
rs9940099
rs8102497
rs11991877
rs80116508
rs7790856
rs76219171
rs6881568
rs2977608
rs79977579
rs16978028
rs7209057
rs6587577
rs117512405
rs10845387
rs188918174
rs869785
rs6536702
rs117407747
rs376641875
rs4498805
rs139669835
rs141214782
rs144204502
rs28577594
rs76666449
rs10112752
rs17445108
rs1907702
rs10773176
rs2538745
rs28363070
rs185174247
rs17803849
rs1003322
rs73730598
rs6054257
rs3891167
rs1291143
rs45604339
rs11769630
rs41269079
rs611646
rs11584821
rs139795227
rs11412296
rs11212631
rs116863223
rs56061761
rs4695407
rs117034449
rs202034370
rs1985369
rs871134
rs9398196
rs4731541
rs145114957
rs4616688
rs10905255
rs10774624
rs66731853
rs12925933
rs450962
rs12369950
All − MR Egger
All − Inverse variance weighted
−2
−1
0
1
2
MR effect size for
'telomere length || id:ieu−b−4879' on 'IDP_dMRI_TBSS_MD_Posterior_thalamic_radiatio
